# High-Pressure Extraction of Antioxidant-Rich Fractions from Shrubby Cinquefoil (*Dasiphora fruticosa* L. Rydb.) Leaves: Process Optimization and Extract Characterization

**DOI:** 10.3390/antiox9060457

**Published:** 2020-05-26

**Authors:** Michail Syrpas, Kiran Subbarayadu, Vaida Kitrytė, Petras Rimantas Venskutonis

**Affiliations:** Department of Food Science & Technology, Kaunas University of Technology, Radvilėnų pl. 19, LT-50254 Kaunas, Lithuania; kiran.subbarayadu@ktu.lt (K.S.); vaida.kitryte@ktu.lt (V.K.); rimas.venskutonis@ktu.lt (P.R.V.)

**Keywords:** *Dasiphora fruticosa*, antioxidant capacity, pressurized-liquid extraction, response surface methodology, phenolic compounds, supercritical carbon dioxide extraction

## Abstract

*Dasiphora fruticosa* (basionym *Potentilla fruticosa*) is a shrub, known in traditional medicine for centuries. Due to the wide range of pharmacological effects, interest and applications of *D. fruticosa* extracts are continually increasing; however, reports on optimization of extraction conditions are scarce. Herein, a multi-step high-pressure extraction process with increasing polarity solvents was developed to isolate valuable fractions from *D. fruticosa* leaves. Supercritical CO_2_ extraction recovered 2.46 g/100 g of lipophilic fraction, rich in polyunsaturated fatty acids. Further, pressurized liquid extractions (PLE) with acetone, ethanol, and water were applied to obtain antioxidant-rich higher polarity extracts. Under optimized PLE conditions, the cumulative polar fraction yield was 29.98 g/100 g. Ethanol fraction showed the highest yield (15.3 g/100 g), TPC values (148.4 mg GAE/g), ABTS^•+^, and DPPH^•^ scavenging capacity (161.1 and 151.8 mg TE/g, respectively). PLE was more efficient than conventional solid–liquid extraction in terms of extraction time, extract yields, and in vitro antioxidant capacity. Phytochemical characterization of PLE extracts by UPLC-Q-TOF-MS revealed the presence of hyperoside, ellagic acid, among other health beneficial phenolic substances. Τhis study highlights the potential of high-pressure extraction techniques to isolate antioxidant-rich fractions from *D. fruticosa* leaves with multipurpose applications, including the prevention and treatment of chronic diseases.

## 1. Introduction

Over the last years, there have been extensive studies on the role of reactive oxygen species in several inflammatory processes and oxidative stress, and their implications to the pathogenesis of degenerative aging diseases, such as atherosclerosis, neurodegenerative illnesses, and cancer [1]. Simultaneously, interest in plant-derived phenolic substances as potential agents in the prevention and treatment of oxidative-stress related disorders has gained significant scientific attention [2]. The screening of various plants has dramatically expanded our knowledge on novel antioxidant substances, their modes of action, and roles, either as protective/prophylactic substances or as therapeutic molecules [3]. Under-investigated aromatic, ornamental flowers and medicinal plants, gained attention for their potential use as sources of nutraceuticals with bioactive properties [4]. A previous study of our group on aromatic and medicinal plants grown in Lithuania revealed that *Dasiphora fruticosa* (basionym *Potentilla fruticosa*) exerts a substantial radical scavenging activity [5,6].

*D. fruticosa,* commonly known as shrubby/bush cinquefoil, is a hardy deciduous flowering shrub. It is native to the cold temperate and subarctic regions of the Northern Hemisphere, often growing at high altitudes in the mountains [6]. In several parts of the world, extract preparations from aerial and underground parts of this genus are traditionally used for their antioxidant, hypoglycemic, anti-inflammatory, anti-tumor, anti-ulcerogenic, and anti-cancer properties, also for the treatment of inflammations, wounds and pathogen infections [7,8]. Besides traditional use, polar extracts from *D. fruticosa* leaves and roots find a large number of applications in the food, cosmetic, and medical industries [9]. The antimicrobial activity of *D. fruticosa* polar extracts against Gram-positive and Gram-negative bacteria, spore-forming bacteria, and fungi has been verified in some previous publications, indicating the potential use of these extracts as natural antimicrobial agents in various food systems [7,8,10,11]. A few years ago, Liu et al. and Wang et al. demonstrated that *D. fruticosa* leaf extracts when combined with green tea polyphenols or *Ginkgo biloba* extracts show synergistic, additive, and antagonistic effects on a variety of oxidation systems [12,13,14]. The reported bioactivity of these extracts is typically attributed to the high phenolic content of this plant. *D. fruticosa,* as several other *Dasiphora* genera, contains a wide range of bioactive substances [8]. Among which, flavonoids and their glycosides, hydrolyzed tannins, sterols, triterpenoids, and phenolic acids [8]. In ornamental flowers and medicinal plants, the content of antioxidant substances is constitutive and known to be influenced primarily by environmental factors [15]. Liu et al. showed that the geographic location, altitude, annual sunshine duration, and temperature, among other factors, influence the qualitative and quantitative phytochemical content of *D. fruticosa* [16,17].

Studies so far focused on the isolation and identification of bioactive substances, primarily based on conventional extraction techniques. However, modern manufacturing practices and consumer trends require the application of extraction methods with reduced environmental impact and use of renewable, non-toxic, cost-effective, readily available, food/pharmaceutical-grade solvents [18]. Supercritical carbon dioxide (SFE-CO_2_) and pressurized liquid extraction (PLE) are extraction techniques that fit all requirements for the green, sustainable recovery of functional components from medicinal plants [19]. Both methods are known for their shorter extraction times, with reduced solvent consumption as compared with conventional fractionation techniques. Automated high-pressure fractionation processes allow obtaining extracts with higher yields, selectivity while reducing the risk of light- or air-induced phytochemical degradation [19].

Although there is an increasing interest in the potential applications and bioactive properties of *D. fruticosa* extracts, to the best of our knowledge, there are no reports on sufficient, sustainable extraction and optimization of extraction parameters of *D. fruticosa*. This study aimed to fill this gap in research and to develop a sequential high-pressure extraction process with increasing polarity solvents to isolate antioxidant-rich extracts from *D. fruticosa* leaves. This approach could be regarded as a sustainable alternative to obtain higher added-value fractions from ornamental and medicinal plants with food, nutraceutical, and pharmaceutical applications.

## 2. Materials and Methods

### 2.1. Plant Material

*Dasiphora fruticosa* samples were collected at blooming stage in the summer of 2018 in the Kaunas Botanical Garden of Vytautas Magnus University, Kaunas, Lithuania (54°52′14″ N/23°54′40″ E). Collected raw materials were air-dried at room temperature (20–25 °C) in a dark, well-ventilated room. The dried leaves were subsequently ground in an ultra centrifugal mill Retsch ZM 200 at 8000 rpm (Retsch GmbH, Haan, Germany) using 0.5 mm sieve. The ground material was stored in hermetically sealed dark glass jars, in a well-ventilated storage place until further extraction and fractionation.

### 2.2. Chemicals and Reagents

Gallic acid (3,4,5-trihydroxybenzoic acid, 99%), 2,2-diphenyl-1-picrylhydrazyl hydrate free radical (DPPH^•^, 95%), 2,2′-azino-bis(3-ethylbenzthiazoline-6-sulphonic acid) (ABTS), 6-hydroxy-2,5,7,8-tetramethylchroman-2-carboxylic acid (Trolox, 97%), Supelco^®^ 37 Component FAME Mix (10 mg/mL in methylene chloride), microcrystalline cellulose (20 μm), catalytic tablet (3.5 g K_2_SO_4_ and 0.4 g CuSO_4_), Na_2_CO_3_, were purchased from Sigma-Aldrich (Steinheim, Germany). Folin–Ciocalteu’s phenol reagent (2 M) was obtained from Fluka Analytical (Bornem, Belgium). NaCl, KCl, KH_2_PO_4_, K_2_S_2_O_8_ were from Lach-Ner (Brno, Czech Republic), Na_2_HPO_4_ from Merck KGaA (Darmstadt, Germany), boron trifluoride 24% methanol solution from Acros organics (Geel, Belgium), ethanol (96.3%, agricultural origin) from Stumbras (Kaunas, Lithuania), liquid nitrogen from AGA SIA (Riga, Latvia), carbon dioxide and nitrogen gases (99.9%) from Gaschema (Jonava region, Lithuania). All solvents for pressurized liquid extraction (PLE) and solid–liquid extraction (SLE) were of analytical grade. Chromatographic analysis was performed using LC-grade grade solvents.

### 2.3. Extraction of Non-Polar and Polar Fractions of D. fruticosa

#### 2.3.1. Supercritical Carbon Dioxide Extraction (SFE-CO_2_)

Extractions were conducted in a pilot-scale supercritical fluid extractor (Applied Separation, Allentown, PA, USA). Briefly, 2.4 ± 0.001 kg of *D. fruticosa* leaves were placed in a 10 L extraction vessel. A surrounding heating jacket maintained the extraction vessel temperature. CO_2_ consumption was measured by a ball float rotameter and a digital mass flow meter in SL/min at standard state: pressure (P) = 100 kPa, temperature (T) = 20 °C, density (ρ) = 0.0018 g/mL. The following conditions were set: extraction pressure and temperature were 45 MPa and 60 °C, respectively. Very similar parameters have been found to ensure a high extraction yield, as per our previous investigations [20,21]. A static extraction time of 30 min was kept followed by 360 min of total dynamic extraction. The extraction yield was determined gravimetrically (±0.001 g) and expressed as g/100 g DW.

#### 2.3.2. Pressurized Liquid Extraction (PLE)

For PLE, 10 ± 0.001 g of *D. fruticosa* leaves were mixed with 10 ± 0.001 g of diatomous earth (100% SiO_2_, Dionex Corporation, Sunnyvale, CA, USA) and placed in 66 mL stainless-steel extraction cells fitted with cellulose filters (Glass Fiber-(X)-Cellulose, Dionex Corporation, Sunnyvale, CA, USA) at both ends. The cells were then placed in an ASE-350 (Thermo Scientific Dionex, Sunnyvale, CA, USA) apparatus. For all extractions, the system pressure was 10.3 MPa with a pre-heat time of 5 min, the cell flush volume was 100%, and the total nitrogen purge time was 120 s. PLE with acetone (PLE-Ac) and ethanol (PLE-EtOH) were optimized by changing temperatures in the range of 60–120 °C and 40–80 °C, respectively, whereas dynamic extraction time was performed in three cycles ranging from 5 to 15 min each. PLE with water (PLE-H_2_O) was performed at 130 °C for 45 min (three cycles × 15 min).

#### 2.3.3. Solid–Liquid Extraction (SLE)

SLE experiments were performed as previously described in the literature with slight modifications [22]. Briefly, 10 ± 0.001 g of *D. fruticosa* leaves and 100 mL of solvent (solid:liquid ratio 1:10) were added in dry glass bottles and placed in a thermostatically controlled shaker (800 rpm) for 360 min. Depending on the solvent, the following temperatures were set: 40 °C for acetone (SLE-Ac), 60 °C for ethanol (SLE-EtOH), and 60 °C for water (SLE-H_2_O). Obtained mixtures were then centrifuged at 6000 g for 10 min, and supernatants were collected and dried.

#### 2.3.4. Soxhlet Extraction

Soxhlet extraction with hexane was performed from 10 ± 0.001 g of *D. fruticosa* leaves. In an automated Soxhlet extractor EZ100H (Behr Labor-Technik, Düsseldorf, Germany), 100 mL of hexane was added and heated under reflux (68 °C at the atmospheric pressure); the rate of extraction—1 cycle/5 min; total extraction time—360 min.

For all extracts, organic solvents were evaporated to dryness in a Büchi V–850 Rotavapor R–210 (Flawil, Switzerland), H_2_O was removed by freeze-drying (−50 °C, 0.5 mbar). The yield was determined gravimetrically and was expressed as g/ 100 g DW (mean values ± standard deviation, n = 2). Non-polar and polar *D. fruticosa* leaves extracts were collected in opaque glass bottles and stored at −20 °C until further analysis. Solid residues after each extraction were dried and kept in a dry, well-ventilated place before SLE, PLE, or the in vitro antioxidant assays.

### 2.4. In Vitro Antioxidant Capacity of D. fruticosa Extracts and Solid Residues

#### 2.4.1. Total Phenolic Content (TPC) by Folin–Ciocalteu’s Assay

TPC of *D. fruticosa* extracts was evaluated by the modified procedure of Singleton et al. [23]. A total of 150 μL of sample or methanol (blank) was mixed with 750 μL of Folin–Ciocalteu’s reagent (1:9, *v*/*v*) and 600 μL of Na_2_CO_3_ solution (75 g/L), left in the dark for 2 h. Absorbance was measured at 760 nm with Spectronic Genesys 8 spectrophotometer (Thermo Spectronic, Rochester, NY). The TPC was expressed as gallic acid equivalents (mg GAE/g extract or DW; mean values ± standard deviation, *n* = 4), employing the dose–response curve for gallic acid (0–80 μg/mL).

#### 2.4.2. The ABTS^•+^ Scavenging Assay

Following Re et al., ABTS^•+^ solution was prepared by mixing 50 mL of ABTS (2 mmol/L PBS (75 mmol/L; pH 7.4)) with 200 μL K_2_S_2_O_8_ (70 mmol/L) and keeping the mixture in the dark at room temperature for 15–16 h before use [24]. To a 1500 μL of working ABTS^•+^ radical solution (0.700 ± 0.010 AU at 734 nm) 25 μL of *D. fruticosa* extract, or methanol (blank) was added, the mixtures were left in the dark for 2 h, and the absorbance was measured at 734 nm with Spectronic Genesys 8 spectrophotometer. TEAC_ABTS_ was expressed as Trolox equivalents (mg TE/g extract or DW; mean values ± standard deviation, *n* = 4), calculated employing dose–response curves for Trolox (0–1500 μmol/L methanol).

#### 2.4.3. The DPPH^•^ Scavenging Assay

The DPPH^•^ scavenging assay was performed following the modified procedure of Brand-Williams et al. [25]. Briefly, to 1000 μL of a ≈89.7 μmol/L (0.800 ± 0.010 AU at 517 nm) DPPH^•^ methanolic solution, 500 μL of *D. fruticosa* extract or methanol (blank) was added. The mixtures were then left in the dark. After 2 h, absorbance was measured at 517 nm with a Spectronic Genesys 8 spectrophotometer. TEAC_DPPH_ was expressed as mg TE/g extract or DW; mean values ± standard deviation, *n* = 4), calculated using dose–response curves for Trolox (0–50 μmol/L methanol).

#### 2.4.4. Antioxidant Capacity Assessment of Solid Substances

Antioxidant capacity of starting plant material and residues after extraction was evaluated by the QUENCHER method [26]. For the Folin–Ciocalteu’s, ABTS^•+^ and DPPH^•^ assays, 10 mg of sample (solid dilutions in microcrystalline cellulose), or cellulose (blank) was used as described elsewhere [27,28]. Data were expressed as mg GAE/g or mg TE/g (mean values ± standard deviation, *n* = 4).

### 2.5. Chromatographic Analysis of D. fruticosa Extracts

#### 2.5.1. Determination of Fatty Acid Composition

The preparation of fatty acid methyl esters (FAME) was performed as previously described [29]. FAME were analyzed on an HRGC 5300 (Mega Series, Carlo Erba, Milan, Italy) gas chromatography system coupled with a flame ionization detector. The analysis was performed using an SP™–2560 (100 m, 0.25 mm (id), 0.20 μm) capillary column (Supelco, Bellefonte, PA, USA). The oven temperature was programmed from 80 to 240 °C with a 4 °C/min ramp and then held at this temperature for 5 min. The injector’s and detector’s temperatures were 220 and 240 °C, respectively. The injection volume was 1 μL, with a split ratio of 100:1. Analyses were performed with helium as a carrier gas at a constant flow rate of 20 mL/s. A standard FAME mixture of 37 fatty acids (C_8_–C_24_) was used for compound identification. Data were presented as percentage ± standard deviation of the total GC-FID peak area of FAME (*n* = 3).

#### 2.5.2. UPLC/ESI-QTOF-MS Analysis of Extracts

Chromatographic analysis of *D. fruticosa* extracts was performed on an Acquity UPLC system (Waters, Milford, USA) coupled to a Bruker maXis UHR-TOF mass spectrometer and photodiode array (PDA) detectors (Bruker Daltonics, Bremen, Germany). The system was equipped with a binary solvent delivery system, an autosampler with a 10 µL sample loop and a temperature-controlled column manager. Chromatographic separation of analytes was achieved with an Acquity BEH C18 column (1.7 µm, 50 × 2.1 mm, i.d.). The solvent protocol and chromatographic conditions were as described previously [27,28]. Tentative compound identification was carried out by comparison of the measured accurate masses and suggested chemical formulas with hits on the Metlin database and previously reported data for *D. fruticosa* extracts. The analysis was performed in triplicate.

### 2.6. Experimental Design

Response surface methodology (RSM) using central composite design (CCD) was employed to determine the effect of the selected independent variables on the PLE extract yields and total phenolic content, and to identify the optimal conditions for PLE with acetone and ethanol. The models were established, and results were analyzed using the software Design-Expert version 12.0.8.0 (Stat–Ease Inc., Minneapolis, MN, USA). All extraction experiments were performed in random order. Statistical significance of the model and each variable was determined using the Student’s *t*-test (*p*-value) at 5% probability level (*p* < 0.05). The adequacy of the model was determined by evaluating the ‘lack of fit’ coefficient and the Fisher test value (F-value) obtained from the analysis of variance.

### 2.7. Statistical Analysis

Mean values and standard deviations were calculated using MS Excel 2016. One-way analysis of the variance (ANOVA), followed by the Tukey’s posthoc test to compare the means that showed significant variation (*p* < 0.05) was performed and calculated using GraphPad Prism software version 7.04 for Windows.

## 3. Results and Discussion

### 3.1. Yields, Antioxidant Capacity, and Fatty Acid Profile of Lipophilic Substances of D. fruticosa Leaves

In the first part of this study, non-polar constituents were isolated from *D. fruticosa* leaves employing supercritical carbon dioxide extraction. SFE-CO_2_ is a non-conventional, high-pressure technique that has already found applications in food and pharmaceutical industries for the recovery of low polarity analytes from various medicinal plants [30]. CO_2_ is an environmentally friendly, non-toxic, non-flammable, and generally recognized as safe (GRAS) solvent [30]. To evaluate the efficiency of SFE-CO_2_, conventional Soxhlet extraction with hexane was chosen as a standard technique [30]. SFE-CO_2_ (45 MPa, 60 °C, 360 min) showed a slightly lower yield (2.46 ± 0.12 g/100 g DW) as compared to Soxhlet extraction with hexane (2.68 ± 0.16 g/100 g DW). However, these differences were not statistically significant (*p* > 0.05). An advantage of SFE-CO_2_ is the production of pure extracts as no further solvent removal is required. On the contrary, maximum allowed solvent residue limits for hexane extracts are strictly regulated (i.e., Directive 2009/32/EC). Generally, the total essential oil or lipophilic product yields from medicinal plants are substantially lower as compared to polar solvent fractions. For example, Miliauskas et al. using tert-butyl methyl ether reported that the non-polar fraction yield (0.26%) from *D. fruticosa* blossoms was significantly lower as compared to ethanol (22.3%) and water fractions (20.3%) that were subsequently prepared [6].

**Figure 1:**
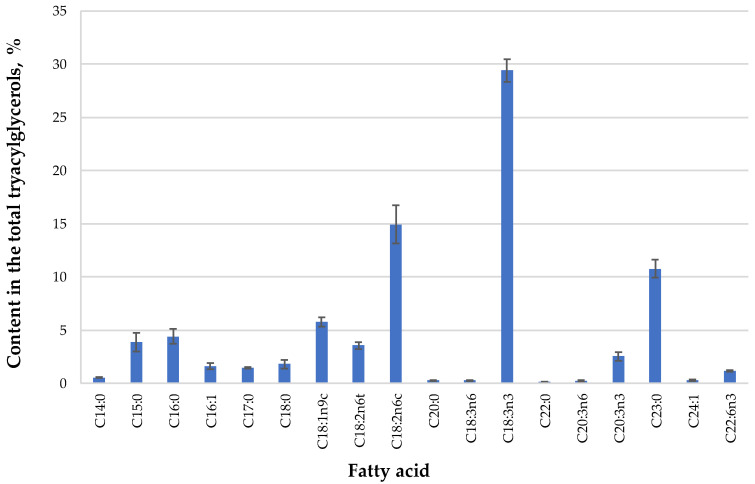
The fatty acid composition of *Dasiphora fruticosa* leaves oil extract extracted by supercritical carbon dioxide (SFE-CO_2_) (45 MPa, 60 °C, 360 min), expressed as percentage ± standard deviation of the total GC-FID peak area of fatty acid methyl esters (FAME) (*n* = 3).

In the next step, SFE-CO_2_ extracts were analyzed by GC-FID to determine the fatty acid composition of triacylglycerols present in the extract (Figure 1). The oil was characterized by the presence of unsaturated fatty acids (total content ≈70% of fatty acids), and more specifically, high contents of linolenic (29.40%), linoleic (14.94%), and oleic (5.79%) acids. Although there are not much data in the literature for the fatty acid profile of *Dasiphora* genera oils, this is in agreement with a previous study, where authors reported that the same fatty acids along with palmitic acid were the major constituents of an SFE-CO_2_ extract of *Potentilla erecta* [31].

Three different assays assessed the in vitro antioxidant capacity of the starting plant material and the defatted fractions: the TPC assay performed with Folin–Ciocalteu’s reagent, the DPPH^•^ scavenging assay, and the ABTS^•+^ decolorization assay (Table 1). However, it should be noted that Folin–Ciocalteu’s reagent is non-specific to phenolic compounds, and it is known to react with a wide range of reducing substances and a series of other groups of compounds. The expression of results as gallic acid equivalents thus provides an estimate of the reducing capacity of the sample and should not be considered as a direct quantitative tool of phenolic compounds.

Typically, the available literature data for *Dasiphora* genera are related to the antioxidant capacity of extractable plant constituents. However, a significant portion of antioxidant substances can be chemically bound to the matrix, which can result in underestimation of the total antioxidant capacity of the sample. To overcome this issue, Gökmen et al. suggested the QUENCHER approach, which evaluates the antioxidant potential of unextracted solid biomaterials and is, in principle, compatible with all the widely utilized in vitro antioxidant capacity assessment protocols [26]. This methodology is useful to evaluate the efficiency of any extraction technique with regards to the recovery of antioxidant components, indicating the necessity of further processing. It may be observed (Table 1) that the effect of SFE-CO_2_ and Soxhlet extraction on TPC, TEAC_ABTS_, and TEAC_DPPH_ values of solid extraction residues in most cases was not significant.

Moreover, in TPC and TEAC_DPPH_ assays, the SFE-CO_2_ residues had even higher antioxidant capacity than the initial material, most likely due to the removal of less active lipophilic compounds or by making bound antioxidants better accessible for reaction in the applied assays. Thus, defatted leaves retained a considerable amount of bioactive compounds with 97%–114%, 91%–97%, and 93%–112% of the initial TPC, ABTS^•+^, and DPPH^•^ scavenging capacity, respectively. Consequently, those fractions remain of great interest for further processing with higher polarity solvents to obtain antioxidant-rich fractions from *D. fruticosa* leaves.

### 3.2. Extraction of Semi-Polar and Polar Constituents from D. fruticosa Leaves

Antioxidant-rich SFE-CO_2_ residue (Table 1) of *D. fruticosa* leaves were further subjected to sequential 3-step PLE with increasing polarity solvents acetone, ethanol, and water in order to isolate semi-polar and polar compounds with potential antioxidant capacity (Supplementary Material, Figure S1). According to the EU Directive 2009/32/EC and European Medicines Agency guidelines for residual solvents EMA/CHMP/ICH/82260/2006, both acetone and ethanol can be used to process raw materials into foodstuffs, food components, or ingredients. These solvents can also be used to produce pharmaceuticals in compliance with a good manufacturing practice. Since the differences in matrix composition and process parameters may significantly influence the effectiveness of the sequential high-pressure extraction, PLE should be optimized for each plant material [19]. Response surface methodology (RSM) with central composite design (CCD) has been extensively applied as a reliable mathematical modeling method to optimize multiple-response processes over the last years. For comparison purposes, isolation of semi-polar and polar fractions from *D. fruticosa* leaves after SFE-CO_2_ was also performed via conventional SLE (Supplementary Material, Figure S1).

#### 3.2.1. PLE-Ac Optimization and Model Analysis

At the 1st step of sequential PLE, acetone was chosen as a solvent. The CCD matrix consisting of 13 experimental runs (four factorial, four axial, and five center points) is presented in Table 2. PLE parameters such as temperature (T = 60–120 °C) and time (τ = 15–45 min) were optimized to isolate antioxidant-rich acetone fractions from the SFE-CO_2_ residue. Two response factors (RF) were taken into consideration, the PLE-Ac yield (RF_I_) and the total phenolic content of the obtained extracts (RF_II_). As reported in Table 2, the yield of acetone-soluble components ranged from 7.05 to 16.47 g whereas the TPC of the obtained extracts ranged from 36.05 to 75.02 mg GAE/g.

Statistical evaluation of the PLE-Ac experimental design is presented in the supplementary material of this manuscript (Supplementary Material, Table S1). The analysis showed that both models were significant (*p* < 0.0001) with non-significant “lack of fit” (*p* > 0.05). Moreover, both models could be considered as reasonably reproducible, as indicated by their coefficients of variation (Supplementary Material, Table S1).

**Figure 2:**
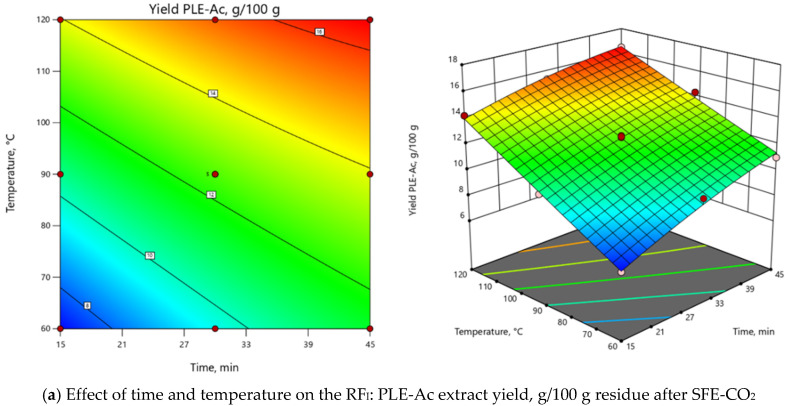

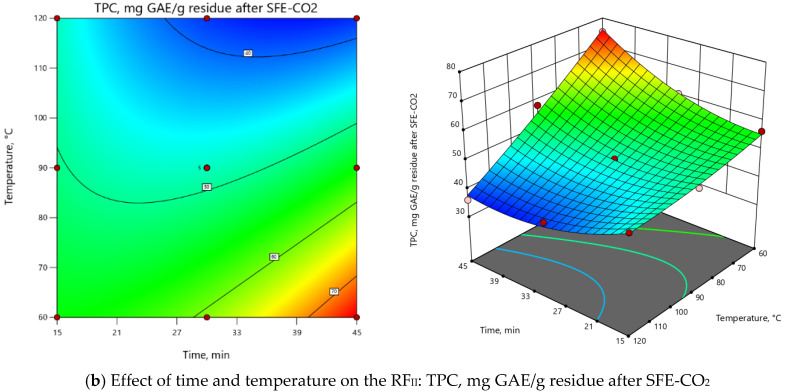
Response surface 3D and 2D plots showing the effects of independent variables on the selected response factors: (**a**) *D. fruticosa* leaves PLE-Ac extract yield (g/100 g residue after SFE-CO_2_); (**b**) total phenolic content (TPC, mg GAE/g residue after SFE-CO_2_).

ANOVA indicated that the extraction temperature (T) was the most influential parameter for both RFs (Supplementary Material, Table S1). As depicted in 3D and 2D response surface plots (Figure 2), change of extraction temperature from 60 to 120 °C exerted dual effects: positive for PLE-Ac extract yield (from 1.5 to 2-fold increase), however negative for TPC (from 1.2 to 2-fold decrease). In the Pareto chart, it can be seen that ≈55%–60% of the observed responses for both RFs derives from the effects of T, while the contribution of other factors is remarkably smaller (Supplementary Material, Figure S2). As presented in Figure 2, the change in PLE time from 15 to 45 min significantly augmented acetone-soluble component yield by 59% and TPC content by 33% at temperatures lower than 65 °C. The prolonged extraction time exerted a less prominent positive effect at the average temperature interval of 65–90 °C (up to 39% and 18% increase in yield and TPC values, respectively), and further reduced TPC by 25% at the maximum PLE temperature levels (Figure 2).

The overall process for both RFs can be summarized in the following second-order polynomial regression equations:

Yield*_PLE-Ac_* = 12.51 + 1.71 × τ + 3.00 × T - 0.42 × τT - 0.32 × τ^2^ + 0.05 × T^2^, (1)

TPC*_PLE-Ac_* = 48.25 + 2.54 × τ – 11.36 ×T – 7.69 × τT + 4.77 × τ^2^ + 1.27 × T^2^, (2)

Considering all observed responses, within the selected range of variables, 62 °C and 45 min were selected as the optimal PLE-Ac conditions to isolate a semi-polar fraction of the highest in vitro antioxidant capacity from *D. fruticosa* leaves. Under this temperature and time combination, 11.67 ± 0.13 g of acetone-soluble substances were recovered from 100 g of plant material residue after SFE-CO_2_, containing 75.96 ± 1.92 mg GAE/g of TPC. When per crude (unextracted) plant material was recalculated, the yield was 11.38 ± 0.12 g/100 g, and the TPC was 74.07 ± 1.87 mg GAE/g DW.

#### 3.2.2. PLE-EtOH Optimization and Model Analysis

Isolation of ethanol-soluble fraction with the highest antioxidant capacity from *D. fruticosa* leaves residue after optimized PLE-Ac (62 °C, 45 min) was set as the main objective for the 2nd step of sequential PLE. For these purposes, the impact of PLE-EtOH temperature T (40–80 °C) and time τ (15–45 min) on two response factors, namely PLE-EtOH yield (RF_I_) and TPC of these extracts (RF_II_), was determined via CCD-RSM (Table 3).

**Figure 3:**
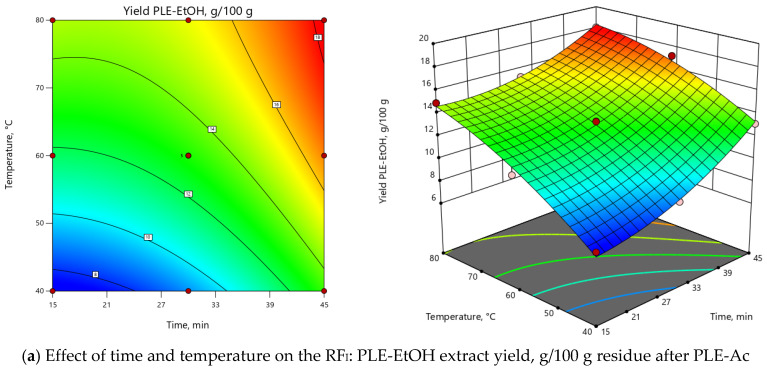

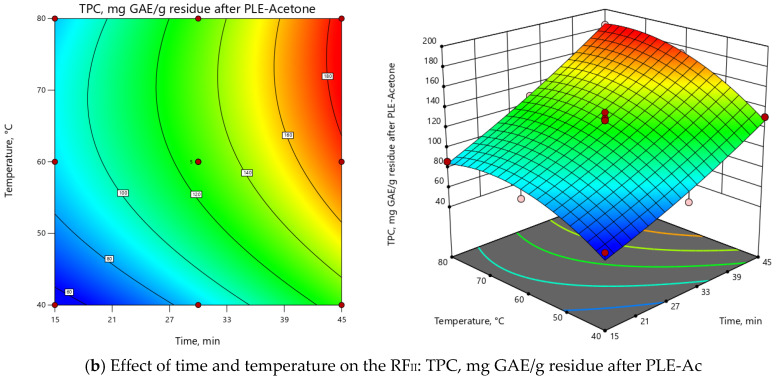
Response surface 3D and 2D plots showing the effects of independent variables on the selected response factors: (**a**) *D. fruticosa* leaves PLE-EtOH extract yield (g/100 g residue after PLE-Ac); (**b**) total phenolic content (TPC, mg GAE/g residue after PLE-Ac).

Different temperature and time combinations yielded 7.52–18.06 g/100 g of ethanol-soluble constituents, while the obtained TPC values were in the range of 75.64–183.46 mg GAE per 1 g of PLE-Ac residue (Table 3). Experimentally obtained and predicted values were in good agreement for both response factors, as confirmed by the determination coefficients R^2^ (RF_I_: 0.99; RF_II_: 0.98), adjusted R^2^ (RF_I_: 0.98; RF_II_: 0.96), and predicted R^2^ (RF_I_: 0.90; RF_II_: 0.85). Based on the ANOVA results, both models were significant (*p* < 0.0001; F_RFI_ = 111.11, F_RFII_ = 60.75) and reproducible (CV of 3.24% and 5.93% for RF_I_ and RF_II_, respectively), also “lack of fit” was not significant with *p* > 0.05 (Supplementary Material, Table S2). 3D and 2D response surface plots (Figure 3) show that the maximum PLE-EtOH yield and TPC values are obtained extracting samples at the high temperature (>75 °C) and prolonged extraction time (45 min) combinations. The main effects of PLE parameters and their interactions are also summarized in the Pareto chart (Supplementary Material, Figure S3): ≈75% of the observed responses for both RFs derives from the effects of T and τ. In comparison, the contribution of other factors is remarkably smaller (<25%).

Second-order polynomial regression equations (in terms of coded factors) for both RFs are the following:

Yield*_PLE-EtOH_* = 13.04 + 2.46 × τ + 3.13 × T - 0.62 × τT + 1.20 × τ^2^ - 0.88 × T^2^, (3)

TPC*_PLE-EtOH_* = 127.22 + 43.64 × τ + 21.93 ×T + 6.52 × τT + 4.57 × τ^2^ – 19.16 × T^2^, (4)

PLE-EtOH under the selected optimal temperature (75 °C) and time (45 min) allowed to recover a substantial portion (17.76 ± 0.38 g/100 g) of ethanol-soluble components with potent in vitro antioxidant capacity (172.29 ± 3.30 mg GAE/g) from the *D. fruticosa* leave residue after PLE-Ac. When per crude (unextracted) plant material was recalculated, these PLE-EtOH yield and TPC values were equal to 15.30 ± 0.33 g/100 g and 148.40 ± 2.84 mg GAE/g DW, respectively.

#### 3.2.3. Comparison of the Extraction Efficiency of PLE and Conventional Techniques

The in vitro antioxidant capacity of solid residue after sequential extractions with CO_2_, acetone, and ethanol was evaluated (Table 4). The results indicate that a small portion of antioxidant substances (10%–15%) remains in the matrix (Table 4). Therefore, the extraction scheme was expanded by introducing an additional step in PLE with a solvent of higher polarity (water). In this case, however, temperature and time optimization for PLE-H_2_O was not performed as (a) the residual plant material activity was low; (b) the measured antioxidant capacity also derives from cell-wall bound substances, which are difficult to extract. Under the tested conditions (130 °C, 45 min), PLE-H_2_O yielded 19.42 ± 0.62 g/100 g of residue after PLE-EtOH or 13.76 ± 0.44g/100 g DW of unextracted leaves. To evaluate the efficiency of PLE, *D. fruticosa* residues after SFE-CO_2_ were also subjected to conventional SLE with acetone, ethanol, and water (Supplementary Material, Figure S1).

Regardless of the applied technique, extraction yields were higher for ethanolic extracts followed by water and then acetone (Table 4). PLE-Ac yield was ≈68% higher than SLE: 11.38 and 6.79 g/100 g DW, respectively. For ethanol, although statistically insignificant (*p* > 0.05), PLE showed a relatively small increase as compared to SLE: 15.30 versus 14.86 g/ 100 g DW. In the last step of the extraction process performed with water as a solvent, the yield was 13.76 and 8.33 g/ 100 g DW for PLE-H_2_O and SLE, respectively (Table 4). Overall, in terms of yield, the total cumulative yield of the polar PLE extracts under optimal conditions was 40.44 g/ 100 g DW (94.2% of the total extracted substances), whereas for the conventional SLE—29.98 g/ 100 g DW (92.4% of the total extracted substances). These results are in line with the previous report of Miliauskas et al., where the polar fraction (ethanol and water in this case) constituted ≈95% of total extractable material [6]. In this study, the application of sequential high-pressure extraction improved the total polar fractions yield by ≈35%. Moreover, the higher efficiency was achieved in significantly shorter extraction times: 135 min in total for the sequential high-pressure extraction as compared to the 1080 min for the SLE. Although there are no previous data for PLE of *Dasiphora* genera, thus direct comparison cannot be made, these findings are in line with previous publications for other medicinal plants. In these cases, too, the application of PLE resulted in significant yield improvement with lower extraction times and solvent consumption [30,32]. Overall, in terms of yield, solvent use, reproducibility, and extraction time, PLE could be recommended as a green alternative to conventional extraction methods.

### 3.3. In Vitro Antioxidant Capacity of Polar D. fruticosa Leaves PLE and SLE Extracts

The in vitro antioxidant capacity of the obtained polar extracts and the solid fractions after each extraction was assessed utilizing TPC by Folin–Ciocalteu’s, the DPPH^•^ scavenging, and the ABTS^•+^ decolorization assays. As reported in Table 4, the TPC in polar extracts ranged from 181.80 to 969.93 mg GAE/g of extract, or 15.14–148.40 mg GAE when recalculated per gram of DW. The highest TPC values were obtained for the ethanolic extracts: 969.93 and 822.38 mg GAE for the PLE-EtOH and the SLE-EtOH, respectively. This fraction was also the most active to scavenge ABTS^•+^ and DPPH^•^, amounting 111.48–166.09 and 96.83–151.81 mg TE/g DW, respectively. Similar observations were also reported in previous studies, where solvent fractionation of biomass or obtained crude extract were performed [6,9]. The obtained values for the SLE-EtOH extract in the DPPH and ABTS assays are in close agreement with previous publications studying ethanolic extracts of *D. fruticosa* [7,33]. Total phenolic content and radical scavenging capacity of acetone and water extracts were lower as compared to ethanol fractions: up to ≈5 and 8-fold in PLE and SLE, respectively. Regardless of the solvent used, the efficiency of PLE to extract antioxidant compounds from *D. fruticosa* leaves was significantly higher as compared to SLE in all assays. In total, 253.83 mg of GAE, 375.68 (ABTS^•+^), and 273.77 (DPPH^•+^) mg of TE were recovered from 1 g of plant material after 135 min of 3-step PLE. Up to 38% lower cumulative TPC (175.71 mg GAE/g), TEAC_ABTS_ (232.96 mg TE/g), and TEAC_DPPH_ (169.59 mg TE/g) values were obtained after sequential SLE in 8-fold longer time (Table 4). The evaluation of the solid fractions with the QUENCHER approach verified the successful recovery of the vast majority (≈98%) of antioxidant substances from *D. fruticosa* leaves. The TPC and TEAC values of residues after final consecutive extraction step were 3.12–3.91 mg GAE and 1.18–3.50 mg TE per 1 g of DW (Table 4). This contributes to only ≈2% of TPC and radical scavenging activity of the initial plant material (Table 1). To the best of our knowledge, this is the first report on the systematic and comprehensive evaluation of the total antioxidant capacity of *D. fruticosa*.3.4. UPLC-ESI-TOF-MS Analysis of *D. fruticosa* Leaves PLE Extracts.

### 3.4. UPLC-ESI-TOF-MS Analysis of D. fruticosa Leaves PLE Extracts

Previous studies have shown that *D. fruticosa* contains a wide range of bioactive substances. Flavonoids and their glycosides, hydrolyzed tannins, sterols, triterpenoids, and phenolic acids have been previously reported [6,7,8,34]. According to the review of Tomczyk and Latté, the high tannin levels, and to a lesser extent triterpenes present in different anatomical parts of *Dasiphora* genera, are responsible for the majority of the reported pharmacological effects both in vitro and in vivo [8]. A preliminary qualitative phytochemical composition of the three different polarity PLE extracts, obtained under optimal conditions reported in this study is presented in Table 5. 

Tentative identification of substances was ascribed based on a comparison of the obtained chromatographic data (suggested formula and accurate mass measurement) with previously identified compounds in the literature of *Dasiphora* genera and available mass spectral databases such as Metlin. In total, 39 out of a total 50 peaks present in the PLE extracts could be tentatively assigned to specific structures (Table 5). For example, the peak eluting at 2.3 min, which was present in all three extracts, showed an experimental *m*/*z* of 463.088 and a suggested deprotonated formula of C_21_H_19_O_12_ could be tentatively ascribed to the quercetin-galactoside (hyperoside). Not surprisingly, many previous studies reported that hyperoside was the predominant, or one of the primary compounds, in various anatomical parts *D. fruticosa*, including leaves [7,33,36]. This flavonoid glycoside has been reported to exert multiple bioactive properties. Anti-inflammatory and anti-redox activities, myocardial protection, and inhibition of osteosarcoma proliferation have been reported for hyperoside [44]. Another substance (R.T 2.3–2.4 min, *m*/*z* = 300.9987, suggested formula C_14_H_5_O_8_), also present in all obtained extracts, can be ascribed to ellagic acid, a commonly reported bioactive substance of *D. fruticosa*. Epidemiological studies indicate that intake of foods that are rich in ellagic acid or ellagitannins, which under physiological conditions in vivo are hydrolyzed to ellagic acid, can be protective against chronic diseases such as certain types of cancer [45]. Several other structures could be assigned to glycosylated apigenin, quercetin, rhamnetin, and kaempferol (Table 5). Recent epidemiological data indicate health-protective properties against chronic diseases like neurodegenerative, cardiovascular diseases, diabetes, and osteoporosis by the consumption of products rich in dietary flavonoids [46,47]. Except for flavonoids and tannins, the chromatographic analysis also indicated the presence of certain triterpenoids, among which are tormentic acid (*m*/*z* = 487.3431, C_30_H_47_O_5_) and hydroxyursolic acid (*m*/*z* = 471.3483, C_30_H_47_O_4_). Pentacyclic triterpenoids, play a critical role in the management and treatment and of non-communicable diseases like diabetes mellitus, cancer, chronic respiratory, and cardiovascular diseases [48].

## 4. Conclusions

This study outlined the efficacy and potential of high-pressure techniques such as SFE-CO_2_ and PLE for the recovery of antioxidant-rich fractions from *D. fruticosa* leaves. Central composite design and response surface methodology were successfully applied to optimize critical extraction parameters, such as temperature and time. The influence of extraction parameters on process yield and total phenolic content was presented, and the optimal extraction conditions were suggested. The application of sequential high-pressure extraction resulted in higher extraction yields and in significantly shorter times than the conventional alternatives. The in vitro antioxidant capacity of obtained extracts indicated that *D. fruticosa* leaf extracts contain substances with high radical scavenging capacity. Phytochemical characterization of the high-pressure extracts revealed the presence of potent natural antioxidants, such as hyperoside, ellagic acid, and triterpenoids. In conclusion, the results of this study highlight the potential of sequential high-pressure extraction as a simple and efficient alternative for the recovery of natural antioxidants from *D. fruticosa* leaves, a promising source of bioactive compounds for food nutraceutical and pharmaceutical applications.

## Figures and Tables

**Table 1:** Yields (g/100 g), total phenolic content (TPC, mg GAE/g), ABTS^•+^, and DPPH^•^ scavenging capacity (TEAC, mg TE/g) of *D. fruticosa* leaves solid residues after SFE-CO_2_ and Soxhlet extractions.

Sample	Yield, g/100 g DW ^1^	TPC, mg GAE/g DW ^1^	TEAC_ABTS,_ mg TE/g DW ^1^	TEAC_DPPH,_ mg TE/g DW ^1^
Starting plant material	-^na^	200.57 ± 4.30 ^a^	255.84 ± 8.65 ^b^	164.50 ± 8.14 ^a^
Residue after SFE-CO_2_^2^	97.54 *	228.49 ± 5.28 ^b^	247.37 ± 4.43 ^ab^	183.63 ± 5.00 ^b^
Residue after Soxhlet-He ^3^	97.32 *	194.47 ± 3.88 ^a^	232.83 ± 13.43 ^a^	153.38 ± 7.68 ^a^

^1^: Yield, TPC, and TEAC values, expressed per mass unit of unextracted *D. fruticosa* leaves; ^2^: SFE-CO_2_ performed at 45 MPa, 60 °C, 360 min; ^3^: Soxhlet extraction performed at atmospheric pressure (0.1 Mpa), 68 °C, 360 min; *: Yield_solid residue_ = 100−Yield_non-polar extract_, g/100 g DW. He: hexane; SFE-CO_2_: supercritical carbon dioxide extraction; TEAC: Trolox equivalent antioxidant capacity; TPC: total phenolic content. Different superscript letters within the same column indicate significant differences (one-way ANOVA and Tukey’s test. *p* < 0.05).

**Table 2:** Central composite design matrix for the pressurized liquid extraction with acetone (PLE-Ac) optimization of *D. fruticosa* leaves after SFE-CO_2_ (45 MPa, 60 °C, 360 min) and values of observed response factors RF_I_ (PLE-Ac extract yield, g/100 g of residue after SFE-CO_2_) and RF_II_ (TPC, mg GAE/g of pomace residue after SFE-CO_2_).

**Levels and Runs**	**Extraction Variables**		**PLE-Ac Yield,** g/100 g *	**TPC,** mg GAE/g **
	**τ, min**	**T, °C**		
Low level (−1)	15	60		
Medium level (0)	30	90		
Max level (+1)	45	120		
1 (central)	30	90	12.45 ± 0.07	46.41 ± 0.81
2 (central)	30	90	12.61 ± 0.42	47.78 ± 0.60
3 (central)	30	90	12.69 ± 0.12	48.49 ± 0.18
4 (factorial)	30	60	9.98 ± 0.11	60.69 ± 0.50
5 (axial)	15	60	7.05 ± 0.60	56.48 ± 0.37
6 (axial)	45	60	11.00 ± 0.20	75.02 ± 0.27
7 (axial)	45	120	16.47 ± 0.19	36.05 ± 0.05
8 (factorial)	45	90	14.32 ± 0.80	58.15 ± 0.58
9 (axial)	15	120	14.20 ± 0.50	48.29 ± 0.68
10 (central)	30	90	12.54 ± 0.80	48.55 ± 0.63
11 (factorial)	30	120	15.37 ± 0.40	39.69 ± 0.10
12 (factorial)	15	90	10.30 ± 0.39	49.22 ± 0.16
13 (central)	30	90	12.01 ± 0.13	48.71 ± 0.71

*: yields expressed as g/100 g of *D. fruticosa* leaves after SFE-CO_2_ (45 MPa, 60 °C, 360 min); **: TPC expressed as mg GAE/g of *D. fruticosa* leaves after SFE-CO_2_ (45 MPa, 60 °C, 360 min); Ac: acetone; SFE-CO_2_: supercritical carbon dioxide extraction; PLE: pressurized liquid extraction (P = 10.3 MPa); GAE: gallic acid equivalents; TPC: total phenolic content; τ: time; T: temperature.

**Table 3:** Central composite design matrix for the pressurized liquid extraction with ethanol (PLE-EtOH) optimization of *D. fruticosa* leaves after PLE-Ac (10.3 MPa, 62 °C, 45 min) and values of observed response factors RF_I_ (PLE-EtOH extract yield, g/100 g of residue after PLE-Ac) and RF_II_ (TPC, mg GAE/g of pomace residue after PLE-Ac).

Levels and Runs	Extraction Variables		PLE-EtOH Yield, g/100 g *	TPC, mg GAE/g **
	τ, min	T, °C		
Low level (−1)	15	40		
Medium level (0)	30	60		
Max level (+1)	45	80		
1 (central)	30	60	12.90 ± 0.33	130.13 ± 3.16
2 (factorial)	30	40	8.84 ± 0.30	75.64 ± 0.43
3 (central)	30	60	13.37 ± 0.25	125.66 ± 1.60
4 (axial)	15	80	14.94 ± 0.40	86.63 ± 0.49
5 (axial)	15	40	7.52 ± 0.45	60.57 ± 2.50
6 (factorial)	15	60	11.10 ± 1.63	78.92 ± 2.93
7 (central)	30	60	13.01 ± 0.13	127.91 ± 5.71
8 (factorial)	30	80	15.25 ± 0.36	129.03 ± 3.26
9 (axial)	45	40	13.13 ± 0.60	131.33 ± 0.40
10 (central)	30	60	12.83 ± 0.35	135.99 ± 5.73
11 (factorial)	45	60	17.16 ± 0.80	173.20 ± 0.37
12 (central)	30	60	13.32 ± 0.25	127.89 ± 3.95
13 (axial)	45	80	18.06 ± 0.30	183.46 ± 1.19

*: yields expressed as g/100 g of *D. fruticosa* leaves after optimized PLE-Ac (10.3 MPa, 62 °C, 45 min); **: TPC expressed as mg GAE/g of *D. fruticosa* leaves after optimized PLE-Ac (10.3 MPa, 62 °C, 45 min); Ac: acetone; EtOH: ethanol; GAE: gallic acid equivalents; PLE: pressurized liquid extraction (P = 10.3 MPa); TPC: total phenolic content; τ: time; T: temperature.

**Table 4:** Yields (g/100 g), total phenolic content (TPC, mg GAE/g), ABTS^•+^, and DPPH^•^ scavenging capacity (TEAC, mg TE/g) of *D. fruticosa* leaves polar extracts and solid residues after sequential pressurized liquid extraction (PLE) and solid–liquid extraction (SLE) with acetone, ethanol, and water.

Sample	Yield, g/100 g DW ^1^	TPC		TEAC_ABTS_		TEAC_DPPH_	
		mg GAE/g Sample ^2^	mg GAE/g DW ^1^	mg TE/g Sample ^2^	mg TE/g DW ^1^	mg TE/g Sample ^2^	mg TE/g DW ^1^
**Polar extracts (from the residue after SFE-CO_2_)**							
*Sequential high-pressure extraction ^3^:*							
PLE-Ac (62 °C, 45 min)	11.38 ± 0.12 ^c^	650.87 ± 16.45 ^f^	74.07 ± 1.87 ^e^	1402.01 ± 41.39 ^f^	159.55 ± 4.71 ^g^	758.57 ± 16.88 ^f^	86.33 ± 1.92 ^e^
PLE-EtOH (75 °C, 45 min)	15.30 ± 0.33 ^e^	969.93 ± 18.57 ^h^	148.40 ± 2.84 ^g^	1085.59 ± 30.45 ^e^	166.09 ± 4.66 ^g^	992.25 ± 16.01 ^h^	151.81 ± 2.45 ^i^
PLE-H_2_O (130 °C, 45 min)	13.76 ± 0.44 ^d^	227.91 ± 5.05 ^d^	31.36 ± 0.69 ^cd^	363.65 ± 7.34 ^c^	50.04 ± 1.01 ^d^	258.96 ± 6.09 ^d^	35.63 ± 0.84 ^c^
*Sequential conventional extraction ^4^:*							
SLE-Ac (40 °C, 360 min)	6.79 ± 0.02 ^a^	565.62 ± 10.11 ^e^	38.39 ± 0.69 ^d^	1533.87 ± 89.22 ^g^	104.10 ± 6.05 ^e^	837.62 ± 24.76 ^g^	56.85 ± 1.68 ^d^
SLE-EtOH (60 °C, 360 min)	14.86 ± 0.04 ^e^	822.38 ± 23.85 ^g^	122.18 ± 3.54 ^f^	750.34 ± 21.55 ^d^	111.48 ± 3.20 ^e^	651.75 ± 22.41 ^e^	96.83 ± 3.33 ^f^
SLE-H_2_O (60 °C, 360 min)	8.33 ± 0.02 ^b^	181.80 ± 11.65 ^c^	15.14 ± 0.97 ^b^	208.75 ± 10.90 ^b^	17.38 ± 0.91 ^b^	191.08 ± 4.82 ^c^	15.91 ± 0.40 ^b^
**Solid fractions (from the residue after SFE-CO_2_):**							
*Sequential high-pressure extraction ^3^:*							
Residue after PLE-Ac	86.16 *	187.11 ± 9.68 ^c^	161.21 ± 8.34 ^h^	149.41 ± 5.82 ^b^	128.73 ± 5.02 ^f^	136.91 ± 11.18 ^b^	117.96 ± 9.63 ^h^
Residue after PLE-Ac-EtOH	70.86 **	36.17 ± 1.98 ^b^	25.57 ± 1.40 ^c^	43.46 ± 1.69 ^a^	30.79 ± 1.20 ^c^	23.40 ± 0.62 ^a^	16.58 ± 0.44 ^b^
Residue after PLE-Ac-EtOH-H_2_O	57.10 ***	5.46 ± 0.03 ^a^	3.12 ± 0.02 ^a^	2.34 ± 0.12 ^a^	1.34 ± 0.07 ^a^	2.07 ± 0.17 ^a^	1.18 ± 0.10 ^a^
*Sequential conventional extraction ^4^:*							
Residue after SLE-Ac	90.75 *	177.20 ± 5.28 ^c^	160.81 ± 4.79 ^h^	136.39 ± 1.24 ^b^	123.77 ± 1.12 ^f^	117.82 ± 6.26 ^b^	106.92 ± 5.68 ^g^
Residue after SLE-Ac-EtOH	75.89 **	34.55 ± 0.59 ^b^	26.22 ± 0.45 ^c^	25.67 ± 1.52 ^a^	19.48 ± 1.15 ^b^	22.59 ± 2.07 ^a^	17.15 ± 1.57 ^b^
Residue after SLE-Ac-EtOH-H_2_O	67.56 ***	5.79 ± 0.06 ^a^	3.91 ± 0.04 ^a^	3.50 ± 0.27 ^a^	2.36 ± 0.18 ^a^	2.32 ± 0.21 ^a^	1.57 ± 0.14 ^a^

^1^: Yield, TPC, and TEAC values, expressed per mass unit of unextracted *D. fruticosa* leaves; ^2^: TPC and TEAC values, expressed per mass unit of extract or solid residue after each step of extraction; ^3,4^: PLE experiments were conducted at 10.3 MPa pressure, SLE - at atmospheric pressure; *: Yield_solid residue_ = 100−Yield_Acetone extr._; **: Yield_solid residue_ = 100−(Yield_Acetone extr._ + Yield_EtOH extr._); ***: Yield_solid residue_ = 100−(Yield_Acetone extr._ + Yield_EtOH extr._ + Yield_H2O extr._); Ac: acetone; EtOH: ethanol; PLE: pressurized liquid extraction; SLE: solid–liquid extraction; TEAC: Trolox equivalent antioxidant capacity; TPC: total phenolic content. Different superscript letters within the same column indicate significant differences (one-way ANOVA and Tukey’s test. *p* < 0.05).

**Table 5:** Qualitative phytochemical characterization of *D. fruticosa* leaves semi-polar and polar extracts after sequential pressurized liquid extraction (PLE) with acetone, ethanol, and water.

R.T, min	Meas. Mass, *m/z*	Δ-ppm	Suggested Formula	Adduct	Tentative Identification	PLE Extracts			Reference
						PLE-Ac	PLE-EtOH	PLE-H_2_O	
0.3–0.4	179.0560	0	C_6_H_11_O_6_	M-H	monosaccharide	+	+	+	
0.3–0.4	195.0511	0	C_6_H_11_O_7_	M-H	gluconic acid	-	-	+	
0.3–0.4	215.0325	11	C_12_H_8_O_4_	M-H	bergapten	+	+	-	
0.3–0.4	269.0877	21	C_15_H_9_O_5_	M-H	apigenin	+	-	-	[35]
0.3–0.4	387.1143	14	C_20_H_19_O_8_	M-H	unknown flavonoid	+	+	+	
0.4–0.4	191.0562	0	C_7_H_11_O_6_	M-H	quinic acid	-	+	+	
0.4–0.4	341.1086	0	C_12_H_21_O_11_	M-H	disaccharide	+	+	-	
0.4–0.4	377.0854	6	C_18_H_17_O_9_	M-H	unknown	-	+	-	
0.4–0.5	133.0142	0	C_4_H_5_O_5_	M-H	malic acid	-	-	+	
0.6–0.6	481.0622	0	C_20_H_17_O_14_	M-H	quercetin-glucuronopyranoside	-	-	+	[6,36]
0.7–0.8	191.0198	0	C_6_H_7_O_7_	M-H	citric acid	-	-	+	
0.9–0.9	217.0354	0	C_7_H_3_N_7_O_2_	M-H	unknown	-	-	+	
1.0–1.0	169.0142	0	C_7_H_5_O_5_	M-H	gallic acid	-	-	+	[37,38]
1.6–1.7	483.0784	0	C_20_H_19_O_14_	M-H	digalloyl glycoside	-	+	-	
1.7–1.8	289.0718	0	C_15_H_13_O_6_	M-H	catechin	+	+	+	[6,9]
1.7–1.8	353.0877	0	C_16_H_17_O_9_	M-H	chlorogenic acid	-	+	+	[39]
1.7–1.8	579.1507	0	C_30_H_27_O_12_	M-H	unknown flavonoid glycoside	+	+	-	
1.7–1.8	643.1667	0	C_31_H_31_O_15_	M-H_2_0	kaempferol glycoside	-	+	-	
1.8–1.9	291.0148	1	C_13_H_7_O_8_	M-H	unknown	-	-	+	
1.9–2.0	165.0193	0	C_8_H_5_O_4_	M-H	hydroxybenzoic acid	+	+	-	
1.9–2.0	327.1083	0	C_15_H_19_O_8_	M-H	luteolin-trimethyl ether	+	+	-	[40]
1.9–2.0	577.1347	0	C_30_H_25_O_12_	M-H	apigenin-(coumaroyl)-glucoside (terniflorin)	+	+	+	[41]
1.9–2.0	635.0889	0	C_27_H_23_O_18_	M-H	gallotannin	+	+	-	
1.9–2.0	865.1980	0	C_30_H_26_O_13_	M-H	unknown	+	-	-	
2.2–2.3	576.1272	0	C_30_H_26_O_13_	M-H_2_0	unknown flavonoid glycoside	+	-	-	
2.2–2.3	609.1458	0	C_27_H_29_O_16_	M-H	quercetin-rutinoside (rutin)	+	+	-	[6,36]
2.2–2.3	615.0988	0	C_28_H_23_O_16_	M-H	unknown flavonoid glycoside	+	+	-	-
2.3–2.3	463.0880	0	C_21_H_19_O_12_	M-H	quercetin-galactoside (hyperoside)	+	+	+	[7,9,33,36]
2.3–2.4	300.9987	0	C_14_H_5_O_8_	M-H	ellagic acid	+	+	+	[6,9]
2.3–2.4	477.0674	0	C_21_H_17_O_13_	M-H	quercetin-galacturonide/-glucuronide	+	+	+	
2.5–2.6	319.0456	1	C_15_H_11_O_8_	M-H	dihydromyricetin	-	+	-	
2.6–2.7	433.0771	0	C_20_H_17_O_11_	M-H	quercetin-arabinofuranoside (avicularin)	+	+	-	[41]
2.6–2.7	451.1035	0	C_24_H_19_O_9_	M-H	epigallocatechin-coumarate	+	-	-	
3.1–3.1	485.1094	0	C_24_H_21_O_11_	M-H	epigallocatechin-dimethylgallate	-	+	-	
3.1–3.1	521.0856	15	C_23_H_21_O_14_	M-H	unknown flavonoid glycoside	-	+	-	
3.1–3.1	614.2514	0	C_36_H_38_O_9_	M-H	unknown	+	+	-	
3.1–3.1	720.1593	0	C_38_H_28_N_2_O_13_	M-H	unknown	+	+	-	
3.2–3.2	477.1040	0	C_22_H_21_O_12_	M-H	rhamnetin-glucopyranoside	+	+	+	[6]
3.2–3.2	593.1308	1	C_30_H_25_O_13_	M-H	kaempferol-rutinoside	+	+	+	[6]
3.2–3.3	491.0833	0	C_22_H_19_O_13_	M-H	isorhamnetin-glucuronide	-	-	+	[42]
3.3–3.4	447.0938	1	C_21_H_19_O_11_	M-H	quercitrin/astragalin	+	+	-	[42]
3.3–3.4	628.267	0	C_37_H_40_O_9_	M-H	unknown	+	-	-	
3.9–4.0	329.2336	0	C_18_H_33_O_5_	M-H	tricin/rhamnatin	+	-	-	
5.0–5.1	487.3431	0	C_30_H_47_O_5_	M-H	tormentic acid	+	-	-	[43]
5.0–5.1	533.3489	0	C_30_H_43_N_7_O_2_	M-H	unknown	+	-	-	
5.7–5.8	485.3276	0	C_30_H_45_O_5_	M-H	unknown	+	-	-	
5.7–5.8	531.3332	0	C_31_H_47_O_7_	M-H	unknown	+	-	-	
6.3–6.3	471.3483	0	C_30_H_47_O_4_	M-H	hydroxyursolic acid	+	-	-	[43]
6.3–6.3	517.3537	0	C_31_H_49_O_6_	M-H	unknown	+	-	-	
6.3–6.3	943.7025	1	C_60_H_95_O_8_	M-H	glycerolipid	+	-	-	

+: Detected; -: not detected; Ac: acetone; EtOH: ethanol; PLE: pressurized liquid extraction.

## Supplementary Materials

The following are available online at https://www.mdpi.com/2076-3921/9/6/457/s1. Table S1: Analysis of variance of the regression parameters for the PLE-Ac response surface quadratic model of *D. fruticosa* leaves for the response factors PLE-Ac extract yield (g/100 g of residue after SFE-CO_2_) and total phenolic content (mg GAE/g of residue after SFE-CO_2_). Table S2: Analysis of variance of the regression parameters for the PLE-EtOH response surface quadratic model of *D. fruticosa* leaves for the response factors PLE-EtOH extract yield (g/100 g of residue after PLE-Ac) and total phenolic content (mg GAE/g of residue after PLE-Ac). Figure S1: Schematic representation of sequential non-polar and polar constituent isolation from *D. fruticosa* leaves applying high-pressure and conventional extraction techniques. Figure S2: Pareto chart (*p* = 0.05) for the main effects of PLE-Ac temperature (T) and time (τ) and interactions thereof on the total extraction yield (g/100 g residue after SFE-CO_2_) and total phenolic content (TPC, mg GAE/g residue after SFE-CO_2_). Figure S3: Pareto chart (*p* = 0.05) for the main effects of PLE-EtOH temperature (T) and time (τ) and interactions thereof on the total extraction yield (g/100 g residue after PLE-Ac) and total phenolic content (TPC, mg GAE/g residue after PLE-Ac).
