# Supplementary material for: High-Pressure Extraction of Antioxidant-Rich Fractions from Shrubby Cinquefoil (Dasiphora fruticosa L. Rydb.) Leaves: Process Optimization and Extract Characterization
Source: Antioxidants (Basel). 2020 May 26;9(6):457. doi: 10.3390/antiox9060457 (PMC7346160; doi:10.3390/antiox9060457)
Supplement: Supplementary file 1 [file antioxidants-09-00457-s001.pdf]

Article

High-Pressure Extraction of Antioxidant-Rich Fractions from Shrubby Cinquefoil (*Dasiphora fruticosa* L. Rydb.) Leaves: Process Optimization and Extract Characterization

Michail Syrpas*, Kiran Subbarayadu, Vaida Kitrytė and Petras Rimantas Venskutonis [1]

[1] Kaunas University of Technology, Department of Food Science & Technology, Radvilėnų pl. 19, LT-50254 Kaunas, Lithuania; michail.syrpas@ktu.lt (M.S.); kiran.subbarayadu@ktu.lt (K.S.); vaida.kitryte@ktu.lt (V.K.); rimas.venskutonis@ktu.lt (P.R.V.)

* Correspondence: michail.syrpas@ktu.lt

SUPPLEMENTARY MATERIALS

Table S1. Analysis of variance of the regression parameters for PLE-Ac response surface quadratic model of *D. fruticosa* leaves for the response factors PLE-Ac extract yield (g/100 g of residue after SFE-CO_2) and total phenolic content (mg GAE/g of residue after SFE-CO_2)

Source	SS	df	MS	F	*p*-value
**RF I: PLE-Ac extract yield (g/100 g of residue after SFE-CO_2)**					
Model	72.54	5	14.51	109.82	< 0.0001*
Time (τ, min)	17.48	1	17.48	132.30	< 0.0001*
Temperature (T, °C)	54.06	1	54.06	409.26	< 0.0001*
τ T	0.7056	1	0.7056	5.34	0.0541**
τ^2	0.2768	1	0.2768	2.10	0.1910**
T^2	0.0065	1	0.0065	0.0491	0.8310**
Residual	0.9247	7	0.1321		
Lack of fit	0.6403	3	0.2134	3.00	0.1580**
Pure error	0.2844	4	0.0711		
Total SS	73.46	12			
**RF II: TPC (mg GAE/g of residue after SFE-CO_2)**					
Model	1143.36	5	228.67	81.93	< 0.0001*
Time (τ, min)	38.66	1	38.66	13.85	0.0074*
Temperature (T, °C)	774.30	1	774.30	277.41	< 0.0001*
τ T	236.85	1	236.85	84.86	< 0.0001*
τ^2	62.76	1	62.76	22.48	0.0021*
T^2	4.47	1	4.47	1.60	0.2463**
Residual	19.54	7	2.79		
Lack of fit	15.92	3	5.31	5.86	0.0603**
Pure error	3.62	4	0.9056		
Total SS	1162.90	12			

*: significant; **: not significant; PLE: pressurized liquid extraction; RF: response factor; SFE-CO_2: supercritical carbon dioxide extraction; SS: the sum of square; df: the degree of freedom; MS: mean square; F: Fisher value; Ac: acetone; GAE: gallic acid equivalents; TPC: total phenolic content.

Table S2. Analysis of variance of the regression parameters for PLE-EtOH response surface quadratic model of *D. fruticosa* leaves for the response factors PLE-EtOH extract yield (g/100 g of residue after PLE-Ac) and total phenolic content (mg GAE/g of residue after PLE-Ac)

**Source**	**SS**	**df**	**MS**	**F**	***p*-value**
**RF I: PLE-EtOH extract yield (g/100 g of residue after PLE-Ac)**					
Model	101.24	5	20.25	111.11	< 0.0001*
Time (τ, min)	36.46	1	36.46	200.06	< 0.0001*
Temperature (T, °C)	58.66	1	58.66	321.88	< 0.0001*
τ T	1.56	1	1.56	8.54	0.0223*
τ^2	3.98	1	3.98	21.84	0.0023*
T^2	2.16	1	2.16	11.86	0.0108*
Residual	1.28	7	0.1822		
Lack of fit	1.04	3	0.3465	5.87	0.0602**
Pure error	0.2362	4	0.0590		
Total SS	102.52	12			
**RF II: TPC (mg GAE/g of residue after PLE-Ac)**					
Model	15522.75	5	3104.55	60.75	< 0.0001*
Time (τ, min)	11429.32	1	11429.32	223.66	< 0.0001*
Temperature (T, °C)	2885.55	1	2885.55	56.47	0.0001*
τ T	169.91	1	169.91	3.32	0.1110**
τ^2	57.68	1	57.68	1.13	0.3233**
T^2	1013.91	1	1013.91	19.84	0.0030*
Residual	357.71	7	51.10		
Lack of fit	295.30	3	98.43	6.31	0.0536**
Pure error	62.41	4	15.60		
Total SS	15880.47	12			

*: significant; **: not significant; EtOH: ethanol; PLE: pressurized liquid extraction; RF: response factor; SS: the sum of square; df: the degree of freedom; MS: mean square; F: Fisher value; Ac: acetone; EtOH: ethanol; GAE: gallic acid equivalents; TPC: total phenolic content.

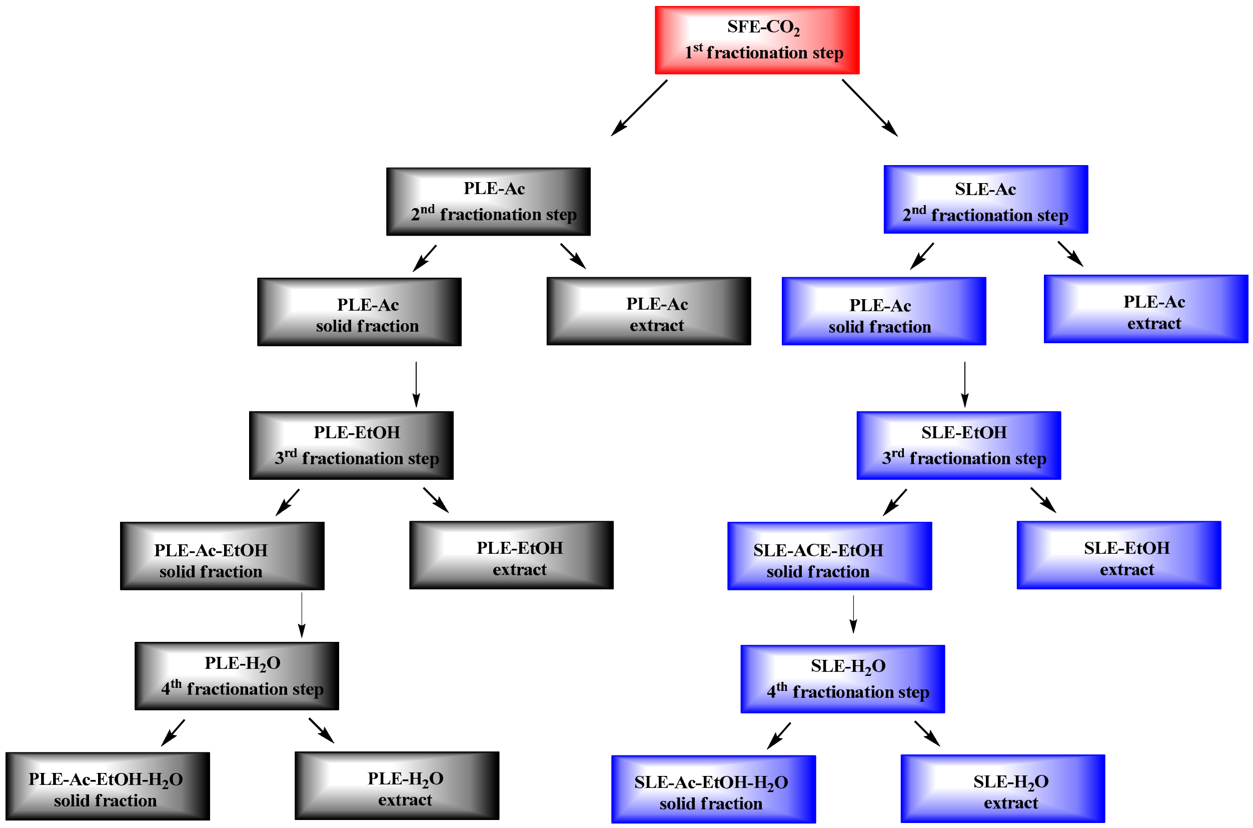

Figure S1. Schematic representation of sequential non-polar and polar constituent isolation from *D. fruticosa* leaves applying high-pressure and conventional extraction techniques
Ac: acetone; EtOH: ethanol; PLE: pressurized liquid extraction; SFE-CO_2: supercritical carbon dioxide extraction; SLE: solid-liquid extraction

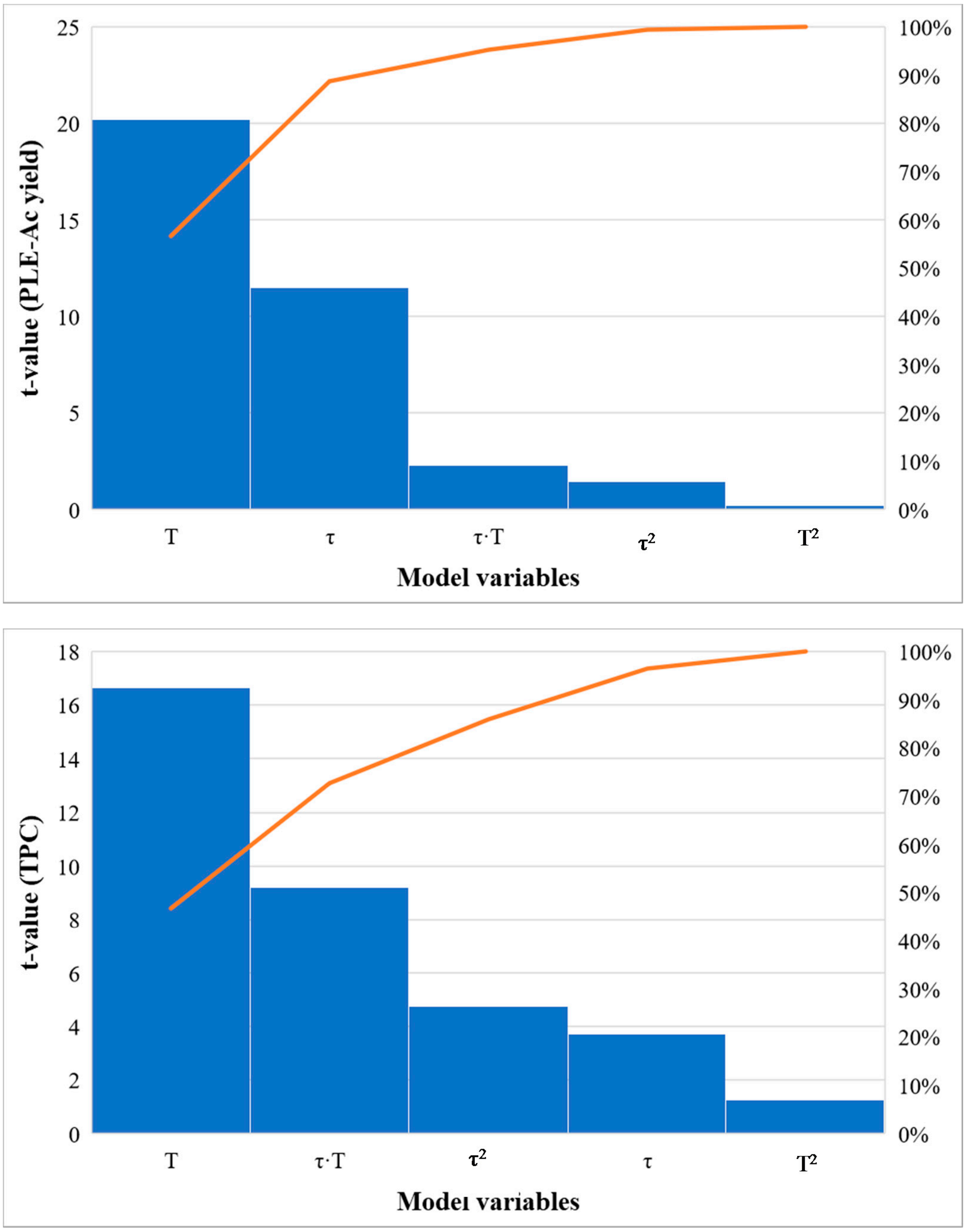

Figure S2. Pareto chart ($p = 0.05$) for the main effects of PLE-Ac temperature (T) and time (τ) and interactions thereof on the total extraction yield (g/100 g residue after SFE-CO_2) and total phenolic content (TPC, mg GAE/g residue after SFE-CO_2)

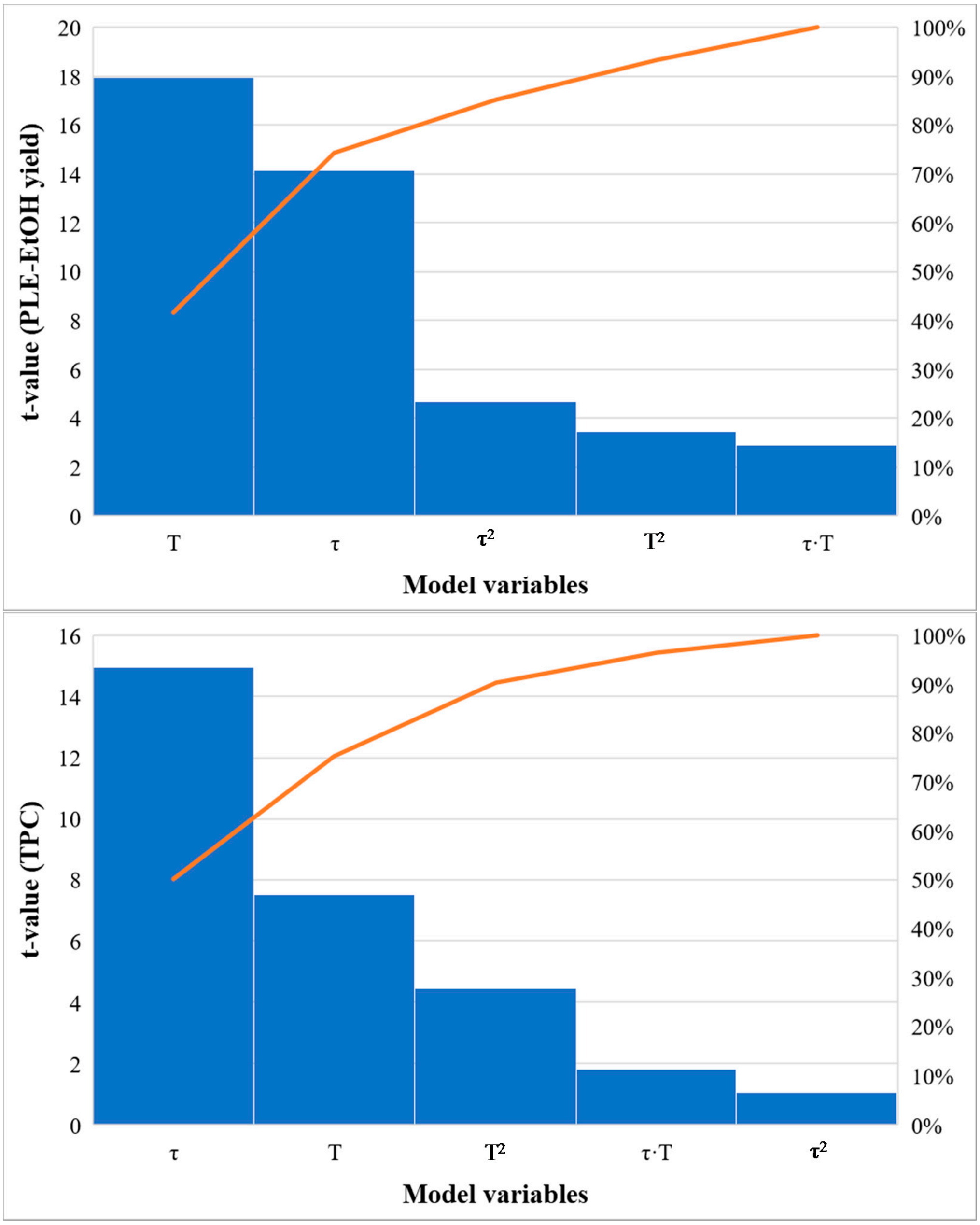

Figure S3. Pareto chart ($p = 0.05$) for the main effects of PLE-EtOH temperature (T) and time (τ) and interactions thereof on the total extraction yield (g/100 g residue after PLE-Ac) and total phenolic content (TPC, mg GAE/g residue after PLE-Ac)
